# Early Physiological Changes Before Rapid Response Team Activation Differentiate Patients Requiring ICU Transfer: A Retrospective Cohort Study

**DOI:** 10.3390/jcm15103722

**Published:** 2026-05-12

**Authors:** Bumin Kim, Sumin Gwon, Gaeun Kim

**Affiliations:** 1 Department of Nursing, Keimyung University Dongsan Hospital, Daegu 42601, Republic of Korea; 2 College of Nursing, Keimyung University, Daegu 42601, Republic of Korea

**Keywords:** rapid response team, clinical deterioration, intensive care unit transfer, early warning, failure to rescue, biomarker change, generalised estimating equations

## Abstract

**Background/Objectives**: Failure to rescue deteriorating ward patients before irreversible organ injury remains a leading cause of preventable in-hospital mortality, yet current rapid response team (RRT) research relies predominantly on cross-sectional comparisons at the moment of activation, overlooking the short-horizon physiological changes that precede it. **Methods**: This retrospective cohort study at a tertiary academic hospital in South Korea included 549 adults (191 ICU-transferred, 358 ward-remaining) with a first RRT activation between September 2023 and August 2025. Generalised estimating equations (GEE) with a time × group interaction modelled differential changes in 12 laboratory variables and the DeepCARS AI-derived risk score between 24 h before activation (T−24 h) and the moment of activation (T0). At T−24 h, physiological profiles were largely similar between groups, indicating that conventional static assessment failed to identify patients destined for ICU transfer. **Results**: Over the ensuing 24 h, patients subsequently transferred to the ICU showed a steeper decline in SpO_2_/FiO_2_ (S/F) ratio (383.4 → 167.1 vs. 369.1 → 260.3; B = −0.547, *p* < 0.001) and steeper increases in lactate (2.91 → 4.02 vs. 2.05 → 2.98 mmol/L; B = 0.154, *p* = 0.045), creatinine (B = 0.076, *p* = 0.038), potassium (B = 0.019, *p* = 0.001), and DeepCARS score (B = 0.073, *p* = 0.028) compared with patients remaining on the ward. All five variables retained significance under Benjamini–Hochberg false discovery rate correction (q < 0.10). Seven inflammatory and haematological markers showed no differential change. Procalcitonin was excluded from the primary analysis because of very high missingness at the pre-activation time point (approximately 75%). **Conclusions**: These findings demonstrate that short-horizon deterioration in oxygenation, perfusion, and renal function—rather than any single earlier measurement—distinguishes patients requiring ICU transfer, supporting the development of change-based early warning criteria to enable earlier clinical escalation.

## 1. Introduction

Failure to rescue—the inability to prevent death or irreversible organ injury once clinical deterioration has begun—accounts for a substantial proportion of preventable in-hospital mortality [1,2]. The rapid response system (RRS) was designed to bridge this gap by detecting deterioration early enough to permit timely intervention [3]. Yet a fundamental question remains inadequately addressed: can the physiological changes that precede deterioration distinguish patients who will require intensive care unit (ICU) transfer from those who can be safely managed on the ward?

The dominant paradigm in rapid response team (RRT) research remains cross-sectional: studies compare patients transferred to the ICU with those remaining on the ward at a single time point—the moment of RRT activation [4,5,6]. This approach identifies who is sicker at activation but cannot answer the clinically more important question of whether impending ICU transfer is detectable earlier. From a clinical decision-making perspective, identifying divergent physiological changes hours before activation is more actionable than characterising severity at the moment of crisis: if the pathways to ICU transfer are distinguishable in advance, escalation can be initiated proactively rather than reactively, potentially reducing failure-to-rescue events.

Two lines of evidence support the feasibility of such early differentiation. First, trend-based analysis of vital signs has been shown to outperform single time-point measurements for predicting clinical deterioration on general wards [1,7,8], establishing that the rate and direction of change carry predictive information beyond absolute values. Second, AI-based early warning systems that generate continuous risk scores from longitudinal data [9,10] may offer the greatest clinical value not through their instantaneous readings, but through their change dynamics across time—information that current escalation protocols do not systematically exploit.

Despite these advances, studies that systematically examine whether serial changes in laboratory biomarkers and AI risk scores before RRT activation can differentiate patients requiring ICU transfer from those safely managed on the ward remain scarce. The limited available evidence suggests that metabolic markers—including lactate, potassium, and inflammation indices—begin to diverge from normal patterns well before clinical recognition [11,12], but whether this principle extends to the full panel of routinely monitored ward parameters has not been tested.

This retrospective cohort study addressed these gaps by testing the hypothesis that short-horizon physiological changes over the 24 h preceding RRT activation can differentiate patients requiring ICU transfer from those safely managed on the ward—even when conventional static assessment at 24 h before activation cannot. Using generalised estimating equations (GEE) to model time × group interactions across 12 routinely available laboratory biomarkers and an institution-specific AI risk score (DeepCARS), we aimed to identify which parameters exhibit differential rates of change associated with subsequent ICU transfer and to evaluate whether change-based assessment provides clinically relevant information beyond single time-point evaluation. We present this study in accordance with the STROBE reporting guideline [13].

## 2. Materials and Methods

### 2.1. Study Design and Setting

This was a single-centre retrospective cohort study at a tertiary academic hospital (≥900 beds) in Daegu, Republic of Korea. The institution operates a 24 h nurse-led RRT that responds to automated alerts from DeepCARS (VUNO Med-DeepCARS^®^, VUNO Inc., Seoul, South Republic of Korea)—a deep learning algorithm that generates a continuous cardiac arrest risk score from vital signs—as well as direct clinician calls. This study was approved by the Institutional Review Board of Keimyung University Dongsan Medical Center (IRB No. DSMC 2025-09-028; approval date: 15 September 2025) and conducted in accordance with the principles of the Declaration of Helsinki. The requirement for informed consent was waived due to the retrospective nature of the study and the use of anonymized electronic health record data. During the study period, there were no formal institutional changes in the RRT activation criteria, RRT workflow, or escalation pathway. The same institutional RRT activation criteria were applied across all departments, without specialty-specific activation thresholds.

### 2.2. Participants

All adult inpatients (≥18 years) on general wards who experienced a first RRT activation between 1 September 2023 and 31 August 2025 were screened. Exclusion criteria were: (1) RRT activated during an ongoing cardiopulmonary resuscitation event; and (2) a do-not-resuscitate (DNR) or treatment-limitation order documented before RRT activation. For patients with multiple RRT activations during a single admission, only the first was analysed.

The analytical cohort was defined based on completed first RRT evaluations documented in the electronic health record. RRT alerts or activations that were cancelled before completion of an RRT evaluation were not separately analysed as a distinct group in this retrospective dataset.

Patients were categorised into two groups based on the clinical decision following RRT evaluation: (1) ICU admission group—patients transferred to an ICU within 24 h of activation; and (2) ward observation group—patients who remained on the general ward. ICU transfer decisions were made through clinical discussion between the RRT and the attending physician, considering the patient’s physiological status, treatment goals, and ICU bed availability.

### 2.3. Data Collection and Time Points

Data were extracted from EHR records by trained research nurses. Two time points were defined for each patient: T0 (RRT activation) and T−24 h (the laboratory result closest to 24 h before T0, within a window of 12–36 h prior). Baseline physiological parameters were collected using a standardized time definition relative to RRT activation: T0 was defined as the time of RRT activation, and T−24 h was defined as the available value closest to 24 h before activation within the prespecified 12–36 h window. Because the pre-activation measurement was defined pragmatically as the value closest to 24 h before activation within a 12–36 h window, the actual sampling interval varied across patients; this variability introduces measurement noise that may attenuate observed differences, biasing results toward the null.

At each time point, the following were recorded: oxygen therapy status; the DeepCARS score; and 12 laboratory parameters—SpO_2_/FiO_2_ ratio (S/F ratio, used as a non-invasive surrogate for PaO_2_/FiO_2_ ratio because arterial blood gas analysis is not routinely performed on general wards), arterial pH, bicarbonate (HCO_3_^−^), lactate, haemoglobin, platelet count, neutrophil-to-lymphocyte ratio (NLR), potassium, creatinine, total bilirubin, C-reactive protein (CRP), and procalcitonin. Vital signs (MAP, HR, RR, body temperature, SpO_2_) were additionally recorded at T0.

### 2.4. Missing Data Handling

Missingness at T−24 h was expected and pre-specified, as laboratory testing in general wards is not standardised. Missingness ranged from 8% to 42% across biomarkers at T−24 h, with highest rates for procalcitonin (75%) and lactate (31%), which are ordered only when sepsis or shock is clinically suspected.

Because procalcitonin had a very high missing rate at T−24 h (approximately 75%), we excluded procalcitonin from the primary GEE analysis and the main multivariable model. Procalcitonin was retained only for descriptive reporting among patients with available measurements, and procalcitonin testing status was considered descriptively as a marker of clinician suspicion for infection or sepsis. For other variables with missing values, multiple imputation was performed under a missing-at-random assumption using fully conditional specification (chained equations) with 10 imputed datasets; predictors included all analysis variables, disposition group, age, and sex; parameter estimates were pooled using Rubin’s rules. Listwise deletion was applied for variables missing in <5%. Complete-case sensitivity analyses were performed to assess the robustness of direction and magnitude of the main interaction effects.

We note that the two-time-point design provides a start-to-end change summary rather than a continuous kinetic profile; the GEE time × group interaction coefficient should be interpreted as a differential rate-of-change estimate between groups.

### 2.5. Statistical Analysis

Descriptive statistics are presented as mean ± SD for continuous variables and frequency with percentage for categorical variables. Univariate between-group comparisons at each time point used independent-samples *t*-test or Mann–Whitney U test for continuous variables and chi-squared or Fisher’s exact test for categorical variables, as appropriate.

To test the primary hypothesis—that physiological changes differed between groups over 24 h—we fitted GEE models for each of the 12 laboratory variables and the DeepCARS score. Procalcitonin was not included as a primary GEE outcome because of its very high missingness at T−24 h. GEE was selected to estimate population-averaged differences in short-horizon change according to ICU disposition, which aligned with the study’s clinical objective of identifying group-level escalation signals rather than subject-specific trajectories; GEE is also robust to missing data under a missing-at-random assumption [14]. The outcome in each model was the log-transformed variable value; the linear predictor included main effects of time (0 vs. −24 h), group (ICU vs. ward), and the time × group interaction term. An exchangeable working correlation structure was specified, which is appropriate given only two repeated measurements per patient. Log-transformation was applied uniformly to all variables for analytical consistency; we note that pH, already a logarithmic scale, showed minimal distributional change after transformation, and its non-significant GEE result (*p* = 0.892) confirms that this choice did not affect substantive conclusions. Normality of the log-transformed variables was confirmed using the Shapiro–Wilk test and Q-Q plot inspection prior to GEE.

The primary estimand of interest was the time × group interaction coefficient (B), representing the differential change from T−24 h to T0 in the ICU group versus the ward group. A significant positive B indicates that the variable rose more steeply in the ICU group; a significant negative B indicates a steeper decline. Given that 12 GEE models were fitted simultaneously, the Benjamini–Hochberg false discovery rate (FDR) procedure was applied to the interaction *p*-values; variables retaining significance at FDR q < 0.10 are reported as such. All analyses were performed with IBM SPSS Statistics version 23.0 (IBM Corp., Armonk, NY, USA). Statistical significance was set at two-sided *p* < 0.05.

## 3. Results

### 3.1. Study Population

**Figure 1:**
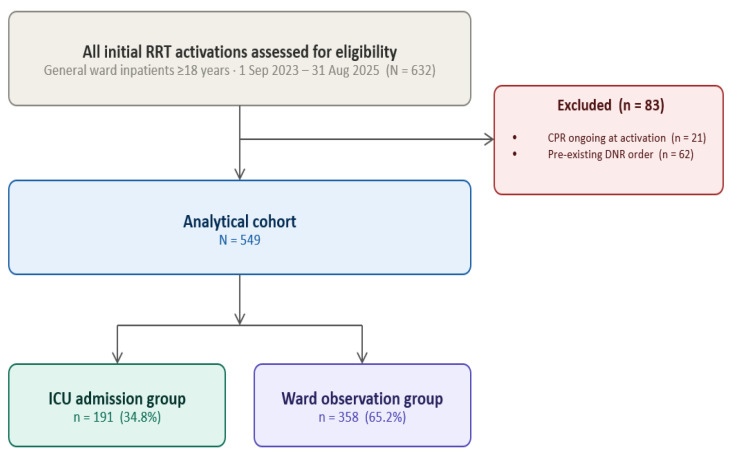
STROBE participant flow diagram. Of 632 patients initially assessed, 83 were excluded (CPR ongoing at activation, *n* = 21; pre-existing DNR order, *n* = 62). The final analytical cohort comprised 549 patients: 191 (34.8%) transferred to the ICU and 358 (65.2%) managed on the ward. CPR, cardiopulmonary resuscitation; DNR, do-not-resuscitate; ICU, intensive care unit; RRT, rapid response team.

A total of 549 patients met the inclusion criteria (191 [34.8%] ICU-transferred; 358 [65.2%] ward-remaining). The study flow is shown in Figure 1. Baseline characteristics and clinical parameters at T0 are presented in Table 1. The ICU group was younger (68.31 ± 14.07 vs. 70.90 ± 12.17 years; *p* = 0.025) and showed significantly worse oxygenation (S/F ratio 167.05 vs. 260.26; *p* < 0.001), lower MAP (*p* < 0.001), higher lactate (4.02 vs. 2.98 mmol/L; *p* = 0.002), and higher procalcitonin > 0.5 ng/mL rate (65.2% vs. 42.9%; *p* = 0.015) at activation. Solid tumour was more prevalent in the ward group (45.8% vs. 27.7%; *p* < 0.001).

### 3.2. Physiological Parameters at 24 h Before RRT Activation (T−24 h)

Table 2 presents parameters at T−24 h. In marked contrast to T0, the two groups were largely similar 24 h before activation, supporting the premise that a single earlier measurement poorly distinguishes later ICU transfer. S/F ratio, lactate, procalcitonin, haemoglobin, platelet count, creatinine, and CRP showed no significant between-group differences at T−24 h.

Only two variables differed significantly at T−24 h. Potassium was higher in the ward group than the ICU group (4.24 ± 0.80 vs. 4.02 ± 0.70 mmol/L; *p* = 0.002). DeepCARS score was also higher in the ward group (75.95 ± 18.57 vs. 70.21 ± 20.83; *p* = 0.002). Oxygen therapy use did not differ significantly (55.0% ward vs. 46.6% ICU; *p* = 0.060).

### 3.3. GEE Analysis: Time × Group Interaction (T−24 h → T0)

**Figure 2:**
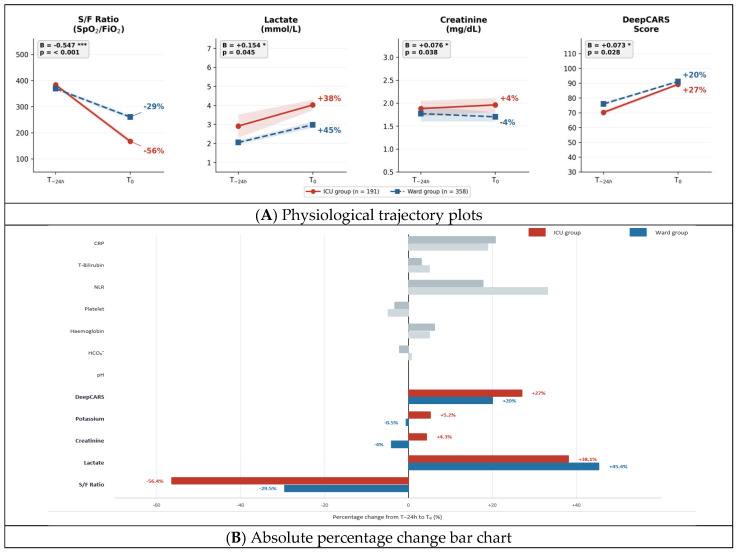
Differential physiological changes and percentage changes from T−24 h to T0, stratified by disposition group (*n* = 549). (**A**) Mean values from 24 h before (T−24 h) to RRT activation (T0) for four of the five biomarkers with significant GEE time × group interactions (S/F ratio, lactate, creatinine, DeepCARS; potassium omitted owing to the small absolute scale of change), stratified by ICU admission group (*n* = 191, red solid line) and ward observation group (*n* = 358, blue dashed line). Points represent group means; shaded bands indicate ±1 standard error of the mean. Regression coefficients (**B**) and significance stars are derived from GEE models: * *p* < 0.05, *** *p* < 0.001. (**B**) Absolute percentage change from T−24 h to T0 for all 12 biomarkers in both groups. Coloured bars (red = ICU, blue = ward) indicate the five GEE-significant variables; grey bars indicate non-significant variables.

GEE results for the time × group interaction are presented in Table 3. Significant time × group interactions were identified for five of 12 parameters (Figure 2; Table 3).

The most pronounced divergence was observed for S/F ratio, which declined steeply in the ICU group (383.4 → 167.1, −56%) compared with the ward group (369.1 → 260.3, −29%) over 24 h (B = −0.547, 95% CI −0.685 to −0.409; *p* < 0.001). Lactate showed a significantly steeper rise in ICU-destined patients (2.91 → 4.02 mmol/L, absolute increase +1.11) versus the ward group (2.05 → 2.98 mmol/L, absolute increase +0.93 mmol/L), with the GEE interaction coefficient confirming a differential change on the log scale (B = 0.154, 95% CI 0.004–0.303; *p* = 0.045). Three additional variables showed significant ICU-group changes: creatinine rose in the ICU group while declining in the ward group (1.88 → 1.96 vs. 1.77 → 1.70 mg/dL; B = 0.076, 95% CI 0.004–0.148; *p* = 0.038); potassium rose 5.2% in the ICU group versus −0.5% in the ward group (4.02 → 4.23 vs. 4.24 → 4.22 mmol/L; B = 0.019, 95% CI 0.008–0.030; *p* = 0.001); and DeepCARS score rose 27% versus 20% (70.21 → 89.14 vs. 75.95 → 91.12; B = 0.073, 95% CI 0.008–0.138; *p* = 0.028). All five variables retained significance under Benjamini–Hochberg FDR correction (q < 0.10).

The remaining seven variables—pH, HCO_3_^−^, haemoglobin, platelet count, NLR, total bilirubin and CRP—showed no significant differential change between groups (all *p* > 0.05; Table 3). These inflammatory and haematological markers evolved in parallel across both groups, suggesting that the distinguishing signal lies specifically in acute oxygen transport, tissue perfusion, and renal function. Procalcitonin was not included in the primary GEE analysis because of its high missingness at T−24 h; among patients with available measurements, procalcitonin was reported descriptively only and was not used for primary inference. Complete-case sensitivity analyses confirmed the direction and statistical significance of all five interaction effects, supporting the robustness of the imputation-based primary analysis.

## 4. Discussion

This study tested whether short-horizon changes in routine laboratory biomarkers and an AI-derived risk score over the 24 h preceding RRT activation can differentiate patients who ultimately require ICU transfer from those safely managed on the ward. To our knowledge, this is the first study to apply a comprehensive change-based approach across the full panel of routine ward laboratory data—including an AI composite score—in a cohort defined by RRT activation. While prior studies have established cross-sectional differences at the moment of activation [4,5,6,15,16], and Churpek et al. [1] demonstrated the superiority of vital-sign trends over single values for deterioration detection, our findings extend this change-based paradigm to laboratory biomarkers and AI-derived scores, with direct implications for clinical escalation decision-making.

The central finding is that these two groups were largely indistinguishable by conventional laboratory parameters 24 h before activation, yet showed markedly different patterns of physiological change in the intervening period. In the ICU-destined group, the 24 h window was marked by progressive oxygenation failure (steep S/F ratio decline), worsening tissue hypoperfusion (rising lactate), and emerging renal dysfunction (rising creatinine and potassium). The observed sequence is biologically compatible with evolving cardiorespiratory or septic deterioration, although the present design does not permit mechanistic attribution at the individual-patient level [17].

The finding that the two groups were almost indistinguishable at T−24 h has direct clinical implications for reducing failure-to-rescue events. It demonstrates that the biological signal distinguishing patients destined for ICU transfer may be largely invisible to conventional static assessment one day before activation—explaining, at least in part, the well-documented phenomenon of delayed RRT activation: when clinicians review 24 h old results, they see a picture that does not yet mandate escalation [18,19,20]. The actionable clinical information lies not in any threshold value at T−24 h, but in the rate and direction of change between T−24 h and T0. Incorporating change-based criteria into existing early warning systems could enable earlier escalation decisions and potentially reduce the interval between physiological deterioration and clinical response.

This interpretation is supported by Churpek et al. [1], who demonstrated that trend-based analysis of vital signs identifies clinical deterioration significantly earlier and more accurately than single time-point values. Our findings extend this principle beyond vital signs to laboratory biomarkers and an AI composite score, suggesting that the change-based paradigm applies across a broader spectrum of routinely monitored ward parameters.

Among the five significant biomarkers, S/F ratio showed the largest and most significant differential decline—falling 56% in the ICU group versus 29% in the ward group (B = −0.547; *p* < 0.001)—consistent with progressive respiratory failure as a primary driver of ICU transfer [21]. The simultaneous lactate rise indicates deepening systemic hypoperfusion accompanying oxygenation failure [22]. Creatinine elevation (+4% vs. −4%; B = 0.076; *p* = 0.038) and hyperkalaemia (+5.2% vs. −0.5%; B = 0.019; *p* = 0.001) emerged in parallel, reflecting the kidney’s vulnerability as a downstream target of circulatory insufficiency [23,24].

The independent change signal of creatinine is particularly noteworthy. Prior RRT studies have reported inconsistent associations between creatinine and ICU transfer at single time points [4,5], and our own T0 comparison confirmed no significant group difference in creatinine at activation. Yet when examined longitudinally, creatinine rise over 24 h was a significant differentiator. This discordance between cross-sectional and longitudinal significance illustrates the fundamental limitation of snapshot analysis for markers that change slowly and reflect cumulative organ stress [25]—precisely the class of markers most relevant to downstream risk.

Two variables were paradoxically higher in the ward group at T−24 h—potassium and DeepCARS score—yet this group had better outcomes. Higher visible potassium and AI alert scores likely triggered proactive clinical responses: earlier laboratory review, medication adjustments, and physician assessment. The ward group’s higher potassium at T−24 h may thus reflect a “treated alert”—a physiological signal that was recognised and addressed before it could cascade. By contrast, the ICU group exhibited subtler initial values that were not initially flagged but subsequently escalated rapidly. This mechanism is consistent with the observation by Xu et al. [26] that hyperkalaemia timing and progression, rather than absolute values, determine prognosis [27].

DeepCARS should be interpreted here as an example of an institution-specific continuously updated composite risk signal rather than as a universally applicable transfer predictor. The DeepCARS trajectory divergence (B = 0.073; *p* = 0.028) indicates that the ICU group’s risk score escalated faster in the final 24 h, even when the absolute value at T−24 h was lower—suggesting the algorithm captures velocity of deterioration, not just level [9,28,29]. Notably, the paradox persisted at T0, where the ward group’s DeepCARS score (91.12) remained marginally higher than the ICU group’s (89.14; *p* = 0.047). This likely reflects the algorithm’s vital-sign-based architecture: ICU-bound patients presented with profoundly low MAP (65.5 vs. 80.1 mmHg) and severe hypoxaemia, a haemodynamic profile that may generate a different risk-score phenotype than the tachycardia-dominant pattern more common in the ward group. This finding reinforces the interpretive caveat that DeepCARS, designed for cardiac arrest prediction, should not be repurposed as a standalone ICU transfer predictor without recalibration.

The absence of significant differential changes for CRP, NLR, and total bilirubin warrants comment. These markers reflect inflammatory and hepatic pathophysiology operating over timescales longer than 24 h; their kinetics of change are too slow to differentiate acute deterioration within this window [30]. This does not diminish their cross-sectional or predictive value at T0, but suggests they are less suitable as short-horizon change markers for early warning systems. Procalcitonin should not be interpreted as a negative finding in this study because approximately 75% of T−24 h values were missing; we therefore excluded it from the primary change-based model and interpreted available procalcitonin data only descriptively. These findings nominate S/F ratio, lactate, creatinine, potassium, and DeepCARS as candidate components for future change-based escalation models, but threshold selection will require prospective derivation and validation.

### Limitations

Several limitations should be acknowledged. First, although we use the term “trajectory” for clinical readability in selected passages, the analysis is based on two clinically available time points and therefore captures short-horizon change rather than fully serial physiological dynamics. Serial measurement at intermediate points (T−12 h, T−6 h) would provide greater temporal resolution and allow identification of inflection points; future studies using continuous vital-sign monitoring or densely sampled laboratory data could define more granular and clinically actionable trajectories.

Second, this is a single-centre retrospective study at a tertiary academic hospital in South Korea, and the findings may not generalise to hospitals without comparable RRT workflows, ward laboratory practices, or institution-specific AI systems such as DeepCARS; multicentre validation across diverse healthcare systems is essential.

Third, T−24 h laboratory data were collected only when tests were routinely ordered, introducing ascertainment bias: patients perceived as sicker may have undergone more frequent or broader laboratory testing, potentially introducing surveillance bias, while patients with missing T−24 h data may have appeared clinically stable, underestimating the change signal. Although multiple imputation was applied, residual informative missingness cannot be excluded.

Fourth, we cannot exclude confounding by treatment intensity in the interval between T−24 h and T0: interventions administered during this period (e.g., fluid resuscitation, supplemental oxygen, diuretics) may have moderated divergence, attenuating the true biological signal.

Fifth, ICU transfer is a clinically determined outcome influenced not only by physiology but also by bed availability, clinician judgment, treatment goals, and local escalation practices; it is not a pure biological endpoint. In addition, RRT alerts or activations that were cancelled before completion of an RRT evaluation could represent a distinct clinical subgroup, but they were not separately analysed in this retrospective dataset. This may introduce potential selection or outcome-classification bias.

Sixth, T−24 h was operationally defined as the laboratory result closest to 24 h before activation within a 12–36 h window; the resulting variability in the actual time interval introduces measurement noise that may have attenuated the observed differences, biasing results toward the null.

Seventh, procalcitonin had a very high missingness rate (approximately 75% at T−24 h). For this reason, procalcitonin was excluded from the primary GEE analysis and the main multivariable model and was reported descriptively only. The high missingness restricts the robustness and interpretability of any procalcitonin-related findings, and procalcitonin should be evaluated in future prospective studies with standardised testing protocols.

Eighth, DeepCARS is validated for cardiac arrest prediction and was applied here as a proxy for overall deterioration risk; its re-purposing for ICU transfer prediction carries interpretive caveats and would require prospective validation.

## 5. Conclusions

In ward patients undergoing RRT activation, subsequent ICU transfer was distinguished not by a single earlier measurement but by the rate and direction of short-horizon physiological change across oxygenation, perfusion, renal, electrolyte, and composite AI-risk domains. Among routinely available variables, S/F ratio, lactate, creatinine, potassium, and DeepCARS showed the clearest differential changes over the 24 h preceding activation. These findings provide an empirical basis for developing change-based early warning criteria that may enable earlier clinical escalation and reduce failure-to-rescue events in rapid response systems.

## Figures and Tables

**Table 1:** Clinical characteristics and laboratory parameters at RRT activation (T0), stratified by disposition group (*n* = 549).

Variables	All Patients (*n* = 549)	ICU Group (*n* = 191)	Ward Group (*n* = 358)	χ^2^ or t	*p*-Value
Demographic and baseline characteristics					
Age, years, mean ± SD	70.00 ± 12.91	68.31 ± 14.07	70.90 ± 12.17	−2.25	0.025
Male sex, *n* (%)	323 (58.8)	108 (56.5)	215 (60.1)	0.643	0.426
Admitting department, *n* (%)					
Medical	376 (68.5)	117 (61.3)	259 (72.3)	7.168	0.028
Surgical	108 (19.7)	47 (24.6)	61 (17.0)		
Other	65 (11.8)	27 (14.1)	38 (10.6)		
Underlying comorbidities, *n* (%)					
Solid tumour	217 (39.5)	53 (27.7)	164 (45.8)	17.213	<0.001
Diabetes mellitus	225 (41.0)	71 (37.2)	154 (43.0)	1.759	0.185
Chronic lung disease	95 (17.3)	20 (10.5)	75 (20.9)	9.557	0.002
Cardiovascular disease	339 (61.7)	118 (61.8)	221 (61.7)	0.001	0.991
Hepatobiliary disease	64 (11.7)	32 (16.8)	32 (8.9)	7.387	0.007
Cerebrovascular disease	139 (25.3)	50 (26.2)	89 (24.9)	0.114	0.735
Chronic kidney disease	109 (19.9)	43 (22.5)	66 (18.4)	1.301	0.254
Solid organ transplantation	20 (3.6)	12 (6.3)	8 (2.2)	5.814	0.016
Clinical characteristics at RRT activation					
Oxygen therapy, *n* (%)	464 (84.5)	177 (92.7)	287 (80.2)	14.878	<0.001
DeepCARS score, mean ± SD	90.44 ± 11.02	89.14 ± 12.40	91.12 ± 10.19	−1.99	0.047
Vital signs					
MAP, mmHg, mean ± SD	—	65.52 ± 39.82	80.09 ± 26.14	—	<0.001
HR, bpm, mean ± SD	108.78 ± 28.69	106.56 ± 28.59	109.78 ± 28.72	—	0.246
SpO_2_, %, mean ± SD	92.57 ± 10.56	90.78 ± 11.49	93.37 ± 10.02	—	0.016
Laboratory parameters					
S/F ratio, mean ± SD	227.83 ± 142.60	167.05 ± 130.15	260.26 ± 138.40	—	<0.001
Lactate, mmol/L, mean ± SD	3.37 ± 3.37	4.02 ± 3.90	2.98 ± 2.95	—	0.002
Haemoglobin, g/dL, mean ± SD	10.58 ± 2.19	11.26 ± 2.01	10.21 ± 2.20	—	0.234
Platelet, ×10^3^/μL, mean ± SD	188.28 ± 125.13	180.86 ± 114.80	192.31 ± 130.38	—	0.309
NLR, mean ± SD	15.44 ± 19.65	14.28 ± 20.60	16.06 ± 19.12	—	0.316
Potassium, mmol/L, mean ± SD	4.23 ± 0.93	4.23 ± 0.99	4.22 ± 0.90	—	0.924
Creatinine, mg/dL, mean ± SD	1.79 ± 1.94	1.96 ± 2.09	1.70 ± 1.86	—	0.134
CRP, mg/dL, mean ± SD	10.72 ± 9.88	9.76 ± 8.58	11.26 ± 10.52	—	0.108
Procalcitonin, *n* (%) †					
≤0.5 ng/mL	64 (49.2)	16 (34.8)	48 (57.1)	5.946	0.015
>0.5 ng/mL	66 (50.8)	30 (65.2)	36 (42.9)		

MAP, mean arterial pressure; HR, heart rate; SpO_2_, peripheral oxygen saturation; S/F ratio, SpO_2_/FiO_2_ ratio; NLR, neutrophil-to-lymphocyte ratio; CRP, C-reactive protein; DeepCARS, deep learning-based continuous cardiac arrest risk score. † Procalcitonin available in 130 patients. MAP is reported separately by group because the overall mean is not clinically meaningful given the significant between-group difference.

**Table 2:** Clinical characteristics and laboratory parameters at 24 h before RRT activation (T−24 h), stratified by disposition group (*n* = 549).

Variables	All Patients (*n* = 549)	ICU Group (*n* = 191)	Ward Group (*n* = 358)	χ^2^ or t	*p*-Value
Oxygen therapy, *n* (%)	286 (52.1)	89 (46.6)	197 (55.0)	3.548	0.060
DeepCARS score, mean ± SD	73.98 ± 19.55	70.21 ± 20.83	75.95 ± 18.57	−2.98	0.002
S/F ratio, mean ± SD	374.09 ± 102.68	383.40 ± 103.28	369.13 ± 101.16	1.55	0.121
pH, mean ± SD	7.41 ± 0.08	7.41 ± 0.08	7.41 ± 0.08	—	0.955
HCO_3_^−^, mEq/L, mean ± SD	24.12 ± 6.16	23.83 ± 4.85	24.27 ± 6.75	—	0.505
Lactate, mmol/L, mean ± SD	2.34 ± 5.16	2.91 ± 8.43	2.05 ± 1.84	—	0.234
Haemoglobin, g/dL, mean ± SD	10.63 ± 6.56	10.64 ± 5.01	10.62 ± 7.26	—	0.966
Platelet, ×10^3^/μL, mean ± SD	195.33 ± 129.72	188.47 ± 144.71	199.02 ± 120.98	—	0.365
NLR, mean ± SD	12.07 ± 15.45	12.00 ± 16.09	12.10 ± 15.11	—	0.942
Potassium, mmol/L, mean ± SD	4.16 ± 0.77	4.02 ± 0.70	4.24 ± 0.80	−3.07	0.002 ‡
Creatinine, mg/dL, mean ± SD	1.81 ± 2.90	1.88 ± 2.36	1.77 ± 3.15	—	0.667
Total bilirubin, mg/dL, mean ± SD	1.23 ± 2.97	1.44 ± 3.33	1.11 ± 2.75	—	0.210
CRP, mg/dL, mean ± SD	8.93 ± 9.92	8.19 ± 9.31	9.34 ± 10.24	—	0.219
Procalcitonin, *n* (%) †					
≤0.5 ng/mL	93 (67.9)	28 (63.6)	65 (69.9)	0.400	0.464
>0.5 ng/mL	44 (32.1)	16 (36.4)	28 (30.1)		

S/F ratio, SpO_2_/FiO_2_ ratio; NLR, neutrophil-to-lymphocyte ratio; CRP, C-reactive protein; DeepCARS, deep learning-based continuous cardiac arrest risk score. † Procalcitonin available in 137 patients at T−24 h. ‡ *p* = 0.002 reflects Mann–Whitney U test (non-normally distributed).

**Table 3:** Generalised estimating equation (GEE) analysis of time × group interaction in physiological parameters from T−24 h to T0 (*n* = 549).

Variable	Effect	B	95% CI LL	95% CI UL	*p*-Value
Significant interaction (time × group)					
S/F ratio	time × ICU	−0.547	−0.685	−0.409	<0.001
Lactate, mmol/L	time × ICU	0.154	0.004	0.303	0.045
Creatinine, mg/dL	time × ICU	0.076	0.004	0.148	0.038
Potassium, mmol/L	time × ICU	0.019	0.008	0.030	0.001
DeepCARS score	time × ICU	0.073	0.008	0.138	0.028
Non-significant interaction					
pH	time × ICU	0.001	−0.016	0.019	0.892
HCO_3_^−^, mEq/L	time × ICU	−0.041	−0.095	0.012	0.128
Haemoglobin, g/dL	time × ICU	0.007	−0.037	0.051	0.757
Platelet, ×10^3^/μL	time × ICU	0.032	−0.064	0.128	0.512
NLR	time × ICU	−0.070	−0.239	0.100	0.419
Total bilirubin, mg/dL	time × ICU	−0.039	−0.126	0.026	0.195
CRP, mg/dL	time × ICU	0.071	−0.130	0.271	0.491
Procalcitonin, ng/mL	time × ICU	0.263	−0.221	0.746	0.287

Note: The procalcitonin row from the previous version of Table 3 has been removed. Procalcitonin was excluded from the primary GEE analysis because of very high missingness at T−24 h (≈75%). B, regression coefficient for the time × group interaction term (log-transformed variables); CI, confidence interval; LL, lower limit; UL, upper limit; S/F ratio, SpO_2_/FiO_2_ ratio; NLR, neutrophil-to-lymphocyte ratio; CRP, C-reactive protein; DeepCARS, deep learning-based continuous cardiac arrest risk score. Positive B: variable rose more steeply in the ICU group over T−24 h → T0. Negative B: variable declined more steeply in the ICU group. All variables log-transformed prior to GEE. Exchangeable working correlation structure used throughout.

## Data Availability

The datasets used and analysed during the current study are available from the corresponding author upon reasonable request.
